# Grey Matter Volume in Substance Use: A Preregistered, Dimensional Approach to Disentangle Substance Use and Disorder Severity

**DOI:** 10.1111/adb.70075

**Published:** 2025-07-28

**Authors:** Kristina Schwarz, Malin K. Hildebrandt, Nele Sauer, Raoul Wüllhorst, Tanja Endrass

**Affiliations:** ^1^ Department of Clinical Psychology and Psychotherapy Technische Universität Dresden Dresden Germany

**Keywords:** addiction, dimensional, grey matter volume, substance‐related problems, ventromedial prefrontal cortex

## Abstract

This preregistered study investigates whether altered grey matter volume (GMV) in the insula and ventromedial prefrontal/anterior cingulate cortex (vmPFC/ACC) ‐ regions commonly implicated in substance use disorder (SUD) ‐ is associated with the degree of substance use or with the severity of substance‐related problems, two distinct but correlated facets of SUD. Baseline structural MRI and behavioural assessment of substance use, substance‐related problems (i.e., DSM‐5 disorder severity) and negative urgency were conducted in 134 (poly‐)substance users. At 1‐year follow‐up, behavioral assessments were repeated in 120 participants. Linear regression analyses tested associations between GMV in predefined regions (insula, vmPFC and ACC) and (1) degree of use, (2) substance‐related problems and (3) substance‐related problems controlled for use. Mediation analyses tested whether negative urgency mediated the problem‐specific associations. GMV in all regions negatively related to substance‐related problems and use (*p*_*BH*_ < 0.05). Controlled for use, GMV in the insula and vmPFC (*p*_*BH*_ < 0.05) but not ACC (*p*_*BH*_ = 0.06) related to substance‐related problems. Follow‐up results revealed differential patterns, but when controlling for use, GMV reductions at baseline did not significantly relate to follow‐up substance‐related problems (insula: *p*_*BH*_ = 0.06; ACC/vmPFC: *p*_*BH*_ > 0.23). Negative urgency related to GMV in the vmPFC (*p*_*BH*_ = 0.02) and mediated the association between vmPFC volume and substance‐related problems controlled for use (indirect effect: CI [−0.12, −0.02]). We demonstrate that smaller GMV in the vmPFC and insula specifically relates to substance‐related problems beyond substance use, albeit with distinct predictive value for prospective symptom development. This highlights the importance of distinguishing between the two facets of SUD to understand why some substance users develop SUD.

## Abstract 2

This preregistered study investigated grey matter volume (GMV) in polysubstance users. GMV in the insula and ventromedial prefrontal cortex was specifically linked to substance‐related problems (i.e. addiction severity), independent of the degree of substance use. Negative urgency mediated the relationship between ventromedial prefrontal cortex volume and substance‐related problems (controlled for degree of use). These findings underscore the importance of distinguishing between substance use and problem severity to improve understanding of risk factors and inform targeted interventions.

## Graphical abstract

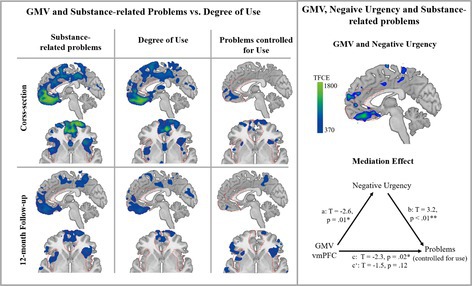

## 1 Introduction

Substance use disorder (SUD) is one of the most prevalent mental disorders with detrimental effects on individuals' physical health, mental well‐being and quality of life. It is linked to chronic diseases, impaired cognition and disruptions in personal and occupational functioning [1]. However, not all substance users develop SUD, defined by characteristic substance‐related problems such as loss of control or neglecting social roles [2]. Although the degree of use is strongly correlated with substance‐related problems [3], some individuals exhibit high degrees of use with few substance‐related problems, whereas others experience significant problems despite low levels of use [3, 4]. Studies on SUD typically compare individuals diagnosed with SUD to healthy controls, where groups differ in both the degree of substance use and substance‐related problems. Hence, the specific associations of potential risk factors remain unclear. Recent studies highlight the potential of disentangling these two facets of SUD [3, 5, 6] to better understand the mechanisms of addiction and develop more personalized interventions. For example, genetic studies have revealed distinct genetic influences on measures of use versus problematic use [7]. Similarly, we previously showed that hypoactivation in the inferior frontal gyrus during inhibitory control was specifically related to substance‐related problems, whereas hyperactivation in the same region was associated specifically with the degree of use, suggesting that the two facets may have dissociable and even opposing effects [6]. However, the question of whether grey matter volume (GMV) alterations in SUD are related to substance‐related problems (i.e., severity of SUD) or the degree of substance use remains unclear.

GMV alterations are consistently reported in SUD and have been linked to the duration of substance use [8, 9], substance‐related problems and factors influencing the risk and resilience in the development of SUD [8]. Recent meta‐ and mega‐analyses reported diminished GMV in SUD compared to healthy individuals in the bilateral insula extending into inferior frontal and superior temporal cortices and the ventromedial prefrontal cortex (vmPFC) extending into the anterior cingulate cortex (ACC) [10, 11, 12, 13, 14]. A recent mega‐analysis including data of 3240 individuals investigated general and substance‐specific GMV alterations, replicating shared alterations in the insula and vmPFC/ACC, suggesting a lower volume in these regions as a shared neural correlate associated with SUD [10]. Functional changes in this network have been widely reported across substances in drug‐ and non‐drug‐related neural activation paradigms, such as cue exposure, inhibitory control and monetary decision‐making tasks (for a review and meta‐analysis, see [15, 16]). The circuitry model of addiction suggests that the insula, vmPFC and ACC are crucial for the anticipation/preoccupation stage of addiction, which is associated with substance‐related problems like craving, deficits in executive function and impulsivity [17]. Negative urgency (NU)—the tendency to act rashly in response to negative emotions [18]—is pivotal for predicting substance‐related problems [19, 20] and has been associated with structural and functional brain correlates in the network, including the insula, ACC and vmPFC [21]. Although we recently showed that NU relates specifically to substance‐related problems beyond substance use [3], it remains open whether this could account for the problem‐specific association with GMV alterations.

This preregistered MRI study is the first to disentangle the effects of substance‐related problems and the degree of substance use on GMV alterations in a sample of (poly‐)substance users and assess its relation to trait NU. As in our previous study [6], we used a detailed and distinct assessment of both variables, which are often conflated in traditional SUD severity measures such as the Alcohol Use Disorders Identification Test (AUDIT [22]). Therefore, the degree of substance use was assessed through a comprehensive questionnaire on frequency and quantity for each substance used in the past year. The degree of use was operationalized as the total sum of substance‐specific use scores (frequency × subjective quantity). Substance‐related problems were assessed using the SUD section of the SCID‐5 interview, supplemented by severity ratings for each DSM‐5 A‐criterion symptom. Severity scores were assessed separately for each substance, and the sum of substance‐specific ratings was used to operationalize substance‐related problems. Additionally, we assessed substance‐related problems and the degree of use at a 1‐year follow‐up to examine long‐term outcomes.

We hypothesized that, cross‐sectionally and prospectively, regional GMV in the vmPFC, ACC and insula would be negatively related to (1) substance‐related problems, (2) the degree of use and (3) substance‐related problems beyond the degree of use. We further explored whether these problem‐specific GMV alterations are linked to NU.

## 2 Methods

Methods and hypotheses were preregistered and are available in the Open Science Framework (OSF, https://osf.io/9b35a). The hypothesis and analyses investigating the effects of NU were not preregistered and are considered exploratory.

### 2.1 Sample

**FIGURE 1:**
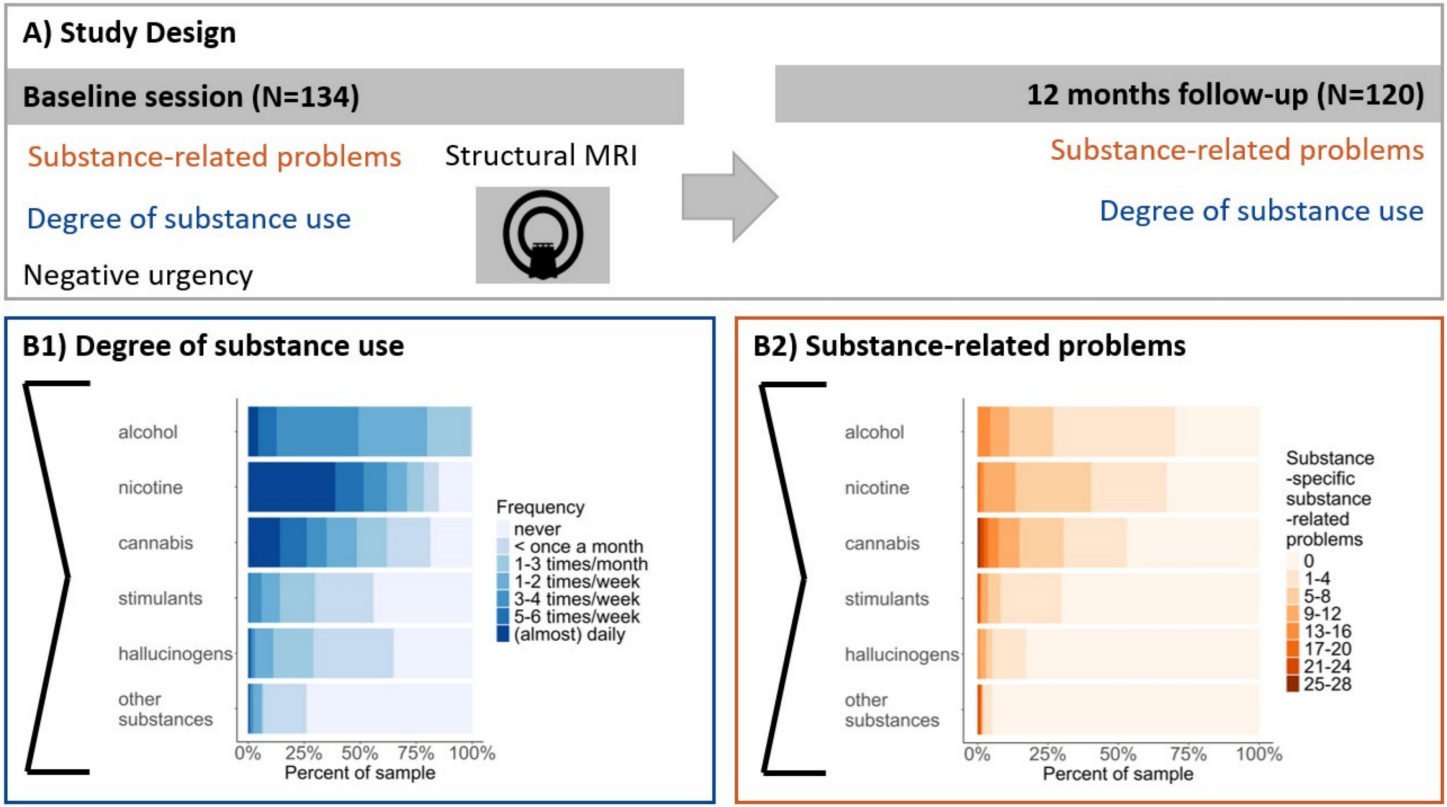
Study procedure and sample characteristics. (A) Study design including baseline and 1 year follow‐up sessions. (B) Distribution of substance‐related problems and degree of substance use. The scores of substance‐specific substance‐related problems reflect the sum of the severity ratings of the DSM‐5 criteria (0 = *not present* to 3 = *extreme*). Overall scores for substance‐related problems and substance use were calculated by summing the substance‐specific scores.

Participants were current substance users recruited from a precursory study [3] and advertisement to ensure a high variability in the degree of substance use. For inclusion, participants had to be current users of at least one psychoactive substance (other than caffeine), with a minimum use frequency of once per month during the preceding year. Participants had to agree to a short‐term abstinence of five plasma half‐lives for all substances to rule out acute substance effects (see Supporting Information for more information on in‐ and exclusion criteria). Mental disorders were assessed using the Structured Clinical Interview for DSM‐5 (SCID‐5 [23]). Comorbidities were permitted, except for acute or severe conditions likely associated with structural brain alterations (e.g., suicidality, eating disorders, ADHD and schizophrenia; see Supporting Information for a full list). Although individuals with a current major depressive disorder (MDD) were excluded, a lifetime MDD diagnosis was allowed and reported by 34% of the sample (Table S1). The final sample consisted of 134 participants (3 diverse, 53 female), 120 of whom (3 diverse, 49 female) completed the follow‐up (see Tables 1 and S1 and Figure 1A). All participants provided written informed consent, and the procedures of this study comply with the Helsinki Declaration and were approved by the local Ethics Committee (approval number: SR‐EK‐270062020).

**TABLE 1:** Sociodemographic, substance use and structural brain characteristics of the participants.

	Laboratory session (*N* = 134)		Follow‐up (*N* = 120)	
Characteristic	M	SD	M	SD
Age	24.7	4.5	24.7	4.4
Years of education	16.2	3.1	16.9	3.2
Degree of substance use	108.3	76.6	93.6	68.2
Substance‐related problems	13.2	11.9	11.2	10.9
VBM parameter				
Total intracranial volume	1564.0	152.4	—	—
Total grey matter volume	747.7	69.1	—	—

### 2.2 Materials

Participants underwent MRI scanning, provided questionnaire data on sociodemographic and substance use measures and were assessed for mental disorders using the structured interview for DSM‐5 (SCID‐5 [23]; see Supporting Information for more information). One year after the laboratory session (range: 12–15 months), participants completed a follow‐up that included the measures of substance use and substance‐related problems (see Table 1). The degree of substance use and substance‐related problems were assessed as previously reported [3, 6]. In short, substance use was assessed using the Dresden Inventory of Substance Use (D‐ISU [6]) including frequency (monthly use occasions) and typical quantity per use occasion (6‐point Likert scale ranging from 0 [*nothing*] to 5 [*very much*]) for each substance used in the past year. The degree of substance use score was calculated as the sum of the substance‐specific use scores (frequency × quantity), representing cumulated drug use over 1 month (data range: 1.25–308.83; see Figure 1B and Supporting Information for more information). Substance‐related problems were assessed by trained clinical psychologists using the SUD section of the SCID‐5 interview [23] for each substance used within the past year, supplemented by severity ratings for each DSM‐5 A‐criterion symptom (scale ranged from 0 [*not present*] to 5 [*extreme*]). Each interview was re‐evaluated by a second independent rater, resulting in excellent interrater reliability (intraclass correlation 0.989). The sum of severity ratings across all substances operationalized substance‐related problems (data range: 0–51; see Figure 1B). For the distribution of substance‐specific severity of SUD in the current sample, see Figure S2. In addition, NU was assessed using the Negative Urgency Subscale of the German version of the 45‐item self‐report UPPS Impulsive Behavior Questionnaire [24]. Items are rated on a 4‐point Likert scale ranging from 1 (*strongly disagree*) to 4 (*strongly agree*) with a total range of 12–48.

### 2.3 Structural MRI Image Acquisition and Preprocessing

All MRI scans were acquired using a 3‐T SIEMENS MAGNETOM TrioTim syngo MR B17 scanner (Siemens, Erlangen, Germany) equipped with a 32‐channel head coil at the Neuroimaging Center of the Technische Universität Dresden. The T1‐weighted whole‐brain images were obtained using a 3D‐MPRAGE sequence (TE/TR = 2.03/2000 ms; voxel size = 1.00 × 1.00 × 1.00 mm^3^, field of view: 208 × 256 × 256 mm with a 100% phase; flip angle = 8°; 208 sagittal slices; TA = 4:54 min; no fat suppression).

Voxel‐based morphometry (VBM) was conducted by using the Computational Anatomy Toolbox (CAT12 [25]) in SPM12 (http://www.fil.ion.ucl.ac.uk/spm/). Preprocessing steps included bias correction, affine registration, tissue segmentation, skull‐stripping and spatial normalization to MNI space using DARTEL, followed by modulation and smoothing. Total intracranial volume (TIV) was estimated for use as a covariate (for more details, see Supporting Information).

### 2.4 Regions of Interest (ROI)

In contrast to the preregistration, we decided to structurally define the regions of interest (ROIs) (ACC, vmPFC and insula) using a well‐established brain atlas (IBASPM Atlas [26]) instead of using masks derived from one meta‐analysis on GMV alterations in SUD. This was done in order to improve the interpretability of results and comparability across studies and enable a more precise and anatomically grounded distinction between areas (see Supporting Information for detailed information and results of the originally preregistered ROIs).

### 2.5 Statistical Analysis

Across all analyses, TIV, age and sex were entered as nuisance variables. Voxel‐wise multiple linear regression analyses implemented in SPSS (IBM, SPSS, Version 40) were performed in two stages: First, we tested whether GMV in the three ROIs predicted substance‐related problems *or* the degree of use in separate regression models. Second, we investigated whether GMV in the three ROIs predicted substance‐related problems while including the degree of use as an additional regressor of interest to investigate the unique variance GMV explained in substance‐related problems. At the whole‐brain level, results were estimated using non‐parametric threshold‐free cluster enhancement (TFCE [27]) implemented in the TFCE toolbox (https://www.neuro.uni‐jena.de/tfce) within CAT12. Significance was set to one‐sided *p* < 0.05 family‐wise error (FWE) correction in whole‐brain analyses and Bonferroni–Holm (BH) [28] correction for the three predefined regions in ROI analyses. Analyses were repeated using the follow‐up measurements. Exploratory analyses focused on the most‐problematic substance instead of poly‐substance problems and use scores to ensure that results were not driven by the aggregation of different substance measures (see details and results in the Supporting Information). Given the high proportion of participants with a lifetime diagnosis of MDD and recent meta‐analytic evidence of GMV alterations in remitted MDD [29], we examined whether problem‐specific GMV alterations in the ROIs were influenced by MDD by including lifetime MDD as an additional covariate using partial regression analyses.

In additional explorative analyses, we tested associations between GMV in the predefined ROIs and NU and (in case of significant associations) tested whether NU mediated the relationship between substance‐related problems (controlled for use) and GMV using the PROCESS macro [30] implemented in SPSS.

## 3 Results

### 3.1 Whole‐Brain Analyses

**FIGURE 2:**
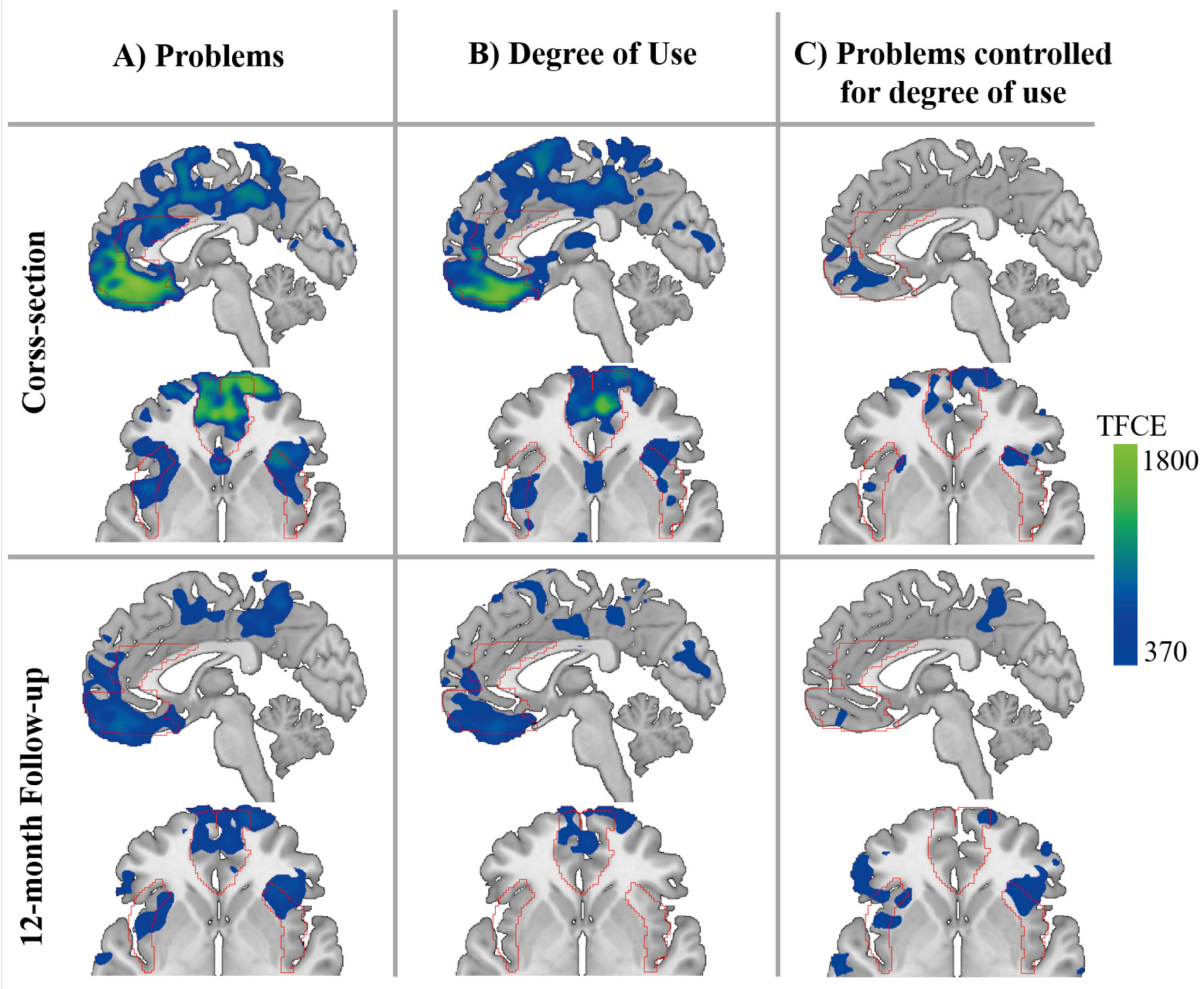
Whole‐brain grey matter volume associations. Local grey matter volumes showing significant negative associations with (A) substance‐related problems, (B) degree of use and (C) substance‐related problems controlled for degree of use, as identified by voxel‐based whole‐brain regression analyses, co‐varying for age, sex, and total intracranial volume (TIV). For illustration, regions of interest are outlined in red.

Exploratory voxel‐wise whole‐brain VBM analyses revealed significant negative associations between substance‐related problems and GMV in multiple areas at a TFCE FWE‐corrected threshold of *p* < 0.05, comprising parts of the left superior and middle frontal gyri as well as the right inferior frontal gyrus, and the left supplementary motor area (see Table S2 and Figure 2A). With respect to the degree of use, we detected a negative association with the GMV in similar regions, including parts of the superior frontal gyrus in both hemispheres and the left supplementary motor area (see Table S2 and Figure 2B). No significant whole‐brain associations between substance‐related problems and regional GMV were detected when additionally controlling for the degree of use (see Table S2 and Figure 2C).

### 3.2 ROI Analyses Investigating Effects of Substance‐Related Problems and Degree of Use

As expected, we detected significant negative associations between GMV and substance‐related problems as well as the degree of use in all ROIs (ACC: *problems*: *p*_*BH*_ = 0.010, B = −0.22, CI [−0.40, −0.04]; *use*: *p*_*BH*_ = 0.036, B = −0.17, CI [−0.36, −0.02]; insula: *problems*: *p*_*BH*_ = 0.004, B = −0.23, CI [−0.38, −0.08]; *use*: *p*_*BH*_ = 0.027, B = −0.17, CI [−0.33, −0.02]; vmPFC: *problems*: *p*_*BH*_ = < 0.001, *B* = −0.43, CI [−0.62, −0.25]; *use*: *p*_*BH*_ = 0.001, B = −0.41, CI [−0.59, −0.22]). Using the follow‐up data, we found that GMV in the insula and vmPFC but not ACC significantly predicted substance‐related problems 1 year later (ACC: *p*_*BH*_ = 0.40, B = −0.08, CI [−0.27, 0.11]; insula: *p*_*BH*_ = 0.024, B = −0.18, CI [−0.33, −0.03]; vmPFC: *p*_*BH*_ = 0.001, B = −0.31, CI [−0.50, −0.11]). In addition, only GMV in the vmPFC, but not ACC or insula, predicted the degree of use 1 year later (ACC: *p*_*BH*_ = 0.31, B = −0.10, CI [−0.31, 0.10]; insula: *p*_*BH*_ = 0.558, B = −0.09, CI [−0.26, 0.08]; vmPFC: *p*_*BH*_ = 0.005, B = −0.32, CI [−0.53, −0.12]). See Figure 2A,B and Tables S3–S14 for details and results using the most‐problematic substance.

### 3.3 ROI Analyses Investigating Effects of Substance‐Related Problems Beyond the Degree of Use

GMV showed a specific negative association with substance‐related problems, controlled for the degree of use, in the insula and vmPFC but not ACC (ACC: *p*_*BH*_ = 0.064, B = −0.11, CI [−0.25, 0.03]; insula: *p*_*BH*_ = 0.025, B = −0.12, CI [−0.24, 0.001]; vmPFC: *p*_*BH*_ = 0.029, B = −0.19, CI [−0.34, −0.03]; see Figure 2C and Tables S15–S17). Results were replicated in the reduced follow‐up sample and using the most‐problematic substance (see Supporting Information). Using the follow‐up data, we found that GMV in none of the ROIs was specifically associated with substance‐related problems beyond use (ACC: *p*_*BH*_ = 0.800, B = −0.02, CI [−0.17, 0.13]; insula: *p*_*BH*_ = 0.063, B = −0.13, CI [−0.25, −0.01]; vmPFC: *p*_*BH*_ = 0.234, B = −0.13, CI [−0.30, 0.03]; see Figure 2C and Tables S18–S20). The inclusion of lifetime MDD as an additional covariate did not alter the overall pattern of results, except that the problem‐specific association in the insula at follow‐up reached significance (see Supporting Information for detailed results).

### 3.4 ROI Analyses Investigating Effects of NU

**FIGURE 3:**
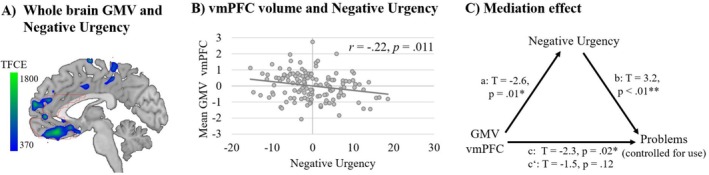
Exploratory analyses of negative urgency, ventromedial prefrontal cortex (vmPFC) volume and substance‐related problems (beyond use). (A) Whole‐brain regression analysis examining the association between grey matter volume (GMV) and negative urgency. For illustration purposes, regions of interest are outlined in red. (B) Partial correlation between mean GMV in the vmPFC and negative urgency. (C) Mediation analysis showing that negative urgency mediates the association between GMV in the vmPFC and substance‐related problems (controlled for degree of use). Age, sex and total intracranial volume (TIV) were included as covariates across all analyses.

NU was associated with GMV in the vmPFC (*p*_*BH*_ = 0.033, B = −1.81, CI [−3.19, −0.43]) but not in the ACC or the insula (*p*_*BH*_ > 0.05; see Figure 3A,B and Tables S21–23). In addition, NU mediated the association between substance‐related problems (controlled for use) and GMV in the vmPFC (indirect effect: CI [−0.12, −0.02]; see Figure 3C and Supporting Information).

## 4 Discussion

This is the first study to examine how GMV differentially relates to substance‐related problems and substance use in poly‐substance users, revealing three main findings. First, we found a negative relationship between GMV in the vmPFC, ACC and insula and both substance use and related problems. Importantly, GMV in the vmPFC and insula was specifically linked to problems, independent of use. Second, prospective associations between baseline GMV and follow‐up substance‐related problems and use patterns differed across ROIs. Whereas vmPFC volume was associated with problems and use at follow‐up, insula volume was only linked to problems, and the ACC showed no associations. However, GMV in the ROIs was not significantly associated with follow‐up substance‐related problems when controlling for use. Third, GMV in the vmPFC was negatively related to NU, which mediated the relationship between vmPFC volume and substance‐related problems, independent of use.

### 4.1 Disentangling Associations Between GMV and Substance‐Related Problems and Use

The observed associations between GMV in the vmPFC, ACC and insula with substance‐related problems as well as the degree of substance use align with recent meta‐ and mega‐analyses on GMV alterations in SUD [10, 11, 12, 13, 14]. Although studies investigating dimensional associations between GMV reductions and SUD characteristics such as problems, drug dosage or duration of drug use are still rare [31, 32, 33], they generally support our results, further highlighting the potential of investigating GMV alterations dimensionally and beyond traditional definitions of health and disease.

However, most of the studies assess SUD severity by mixing the degree of use and problems in one scale (e.g., the AUDIT [22]). This approach can obscure the detection of distinct effects of both SUD facets. By statistically controlling for the degree of use, we demonstrated that GMV reductions in the vmPFC and insula were associated with incremental variance explained by substance‐related problems. This is in line with the circuitry model of addiction, in which the insula and vmPFC are crucial parts of the anticipation/preoccupation stage, associated with core features of substance‐related problems, including craving and impulsivity [17]. The ACC, another key structure in this model, has been linked to substance‐related problems, such as self‐regulation and inhibitory control deficits in SUD [17]. However, although we found that ACC volume was associated with both substance‐related problems and use patterns, no problem‐specific associations emerged. In agreement with studies that directly linked ACC volume to the quantity of substance use (e.g., for alcohol [34]), these findings might suggest that the ACC is more sensitive to changes in substance use behaviours, whereas structural alterations in the insula and vmPFC more specifically reflect substance‐related problems. Importantly, the observed associations remained largely unchanged when statistically controlling for lifetime diagnoses of MDD, suggesting that they are not merely driven by comorbid affective pathology.

### 4.2 The Role of NU on GMV Alterations in the vmPFC

The detected negative association between vmPFC (but not insula) volume and NU might further help to unravel the mechanisms underlying problem‐specific associations with GMV. We examined NU, a trait reflecting impulsivity under distress [18], due to its role in addiction [19, 20], its links to brain alterations in the insula, vmPFC, and ACC [21], and its specific contribution to substance‐related problems beyond use [3]. The significant association between NU and vmPFC volume aligns with a wealth of research [21] such as a recent meta‐analysis linking vmPFC volume to impulsivity [35]. Functionally, the vmPFC is pivotal for higher‐order cognition including decision‐making and self‐regulation [36], which are crucial for managing impulsivity.

We further demonstrate that NU mediated the association between GMV in the vmPFC and substance‐related problems (controlled for use), highlighting its role in explaining variance in vmPFC volume specifically related to substance‐related problems. Supporting this, an influential study showed that cocaine users with SUD (i.e., more substance‐related problems) had decreased vmPFC volumes, whereas recreational users without SUD had increased volumes [37]. These findings underscore the importance of disentangling substance use and substance‐related problems in future research; as such, distinctions may clarify the neural mechanisms underlying SUD symptoms versus use.

Interestingly, NU was associated with GMV in the vmPFC but not with the ACC or insula. Although NU has been linked to functional activation in the ACC and insula, structural findings are rare [21]. The insula is more closely associated with the interoceptive aspects of craving, including the bodily perception of needs or stress, which are closely associated with the experience of craving. In contrast, the ACC is primarily involved in action monitoring and conflict resolution, playing a central role in inhibitory control and self‐regulation deficits often observed in SUD [17]. Although these processes represent key facets of SUD and conceptually overlap with NU, this overlap does not seem to be reflected in the structural alterations observed in the insula and ACC.

### 4.3 Prospective Associations: GMV and Substance‐Related Problems Over Time

The distinction between substance‐related problems and the degree of use, along with the ability to disentangle their neural correlates, underscores a key strength of this study. By incorporating follow‐up data, we provide preliminary insights into the predictive value of GMV reductions for substance‐related outcomes over time. Although not a fully longitudinal design, our findings indicate that associations between GMV reductions and substance‐related problems beyond use might vary over time.

Overall, GMV in the vmPFC was linked to both problems and use, whereas insula volume was associated only with follow‐up problems. Consistent with the notion that the ACC is more directly influenced by substance use, its volume was not prospectively associated with either use or problems. Although GMV in these regions has been linked to substance use [31, 32], their structural plasticity—evident in changes during abstinence or continued use [38]—complicates their interpretation. Notably, none of the ROIs revealed significant problem‐specific effects at follow‐up when controlling for use, although the effect size in the insula remained stable between assessment times (β = −0.2). The drop in significance is due to stricter correction thresholds applied in the Bonferroni–Holm procedure. Although the insula had the second lowest *p* value in the cross‐sectional analyses, it showed the lowest *p* value in the follow‐up tests, leading to a stronger correction of similarly sized effects (uncorrected *p* values: p_baseline_ = 0.025; p_follow‐up_ = 0.021). Interestingly, when additionally controlling for lifetime MDD, the problem‐specific association in the insula at follow‐up reached significance, indicating that prior affective episodes may subtly influence brain–behaviour relationships in this region. Although our findings are speculative and require replication, our results indicate that the insula may play a role in the persistence of substance‐related problems, whereas vmPFC volume appears to predict both use and problems at follow‐up. In contrast, ACC effects may be more sensitive to short‐term dynamics. However, we recognize that our experimental and statistical design is not longitudinal, limiting our ability to infer temporal causality. Future research should include more frequent and repeated MRI assessments to better capture these dynamics.

### 4.4 Limitations

Despite this, the study has several limitations. First, effect sizes of problem‐specific effects were small. Given the moderate correlation between use and problems (*r* = 0.68, *p* < 0.01), small effect sizes were expected and are consistent with findings from our previous fMRI study [6]. Second, correlated measures (i.e., problems and use) can lead to multicollinearity, although control analyses confirmed that the variance inflation factor and tolerance values remained above critical thresholds ([39]; see Supporting Information). Third, although exploratory analyses show that results were not due to aggregating problems and use scores across substances, combining different substance classes may obscure substance‐specific associations. Although this approach accounts for the prevalent poly‐substance use in SUD [40], different substance classes are linked to distinct neurotransmitter systems and brain regions (for a review, see [41]), which may differentially contribute to substance‐related problems and use. Fourth, potential comorbidities such as lifetime MDD might also influence brain–behaviour relationships and obscure subtle substance‐specific effects, underscoring the need for future research to carefully control for psychiatric comorbidities. Future studies should aim to replicate these findings using (1) samples that focus specifically on individuals with high levels of substance use but no SUD diagnosis, and vice versa, and/or (2) larger sample sizes to detect subtle, substance‐specific effects.

## 5 Conclusion

This preregistered study is the first to show that, in a sample of poly‐substance users, substance‐related problems are specifically linked to GMV reductions in the insula and vmPFC, independent of the degree of substance use. We show that these associations display varying levels of temporal stability and are partly mediated by NU. Together with genetic studies and previous work from our group, these findings emphasize the importance of distinguishing mechanisms linked to the degree of use from those related to substance‐related problems and thus addiction severity. This differentiation may help to identify neurobiological markers of addiction risk beyond mere use patterns, supporting more personalized prevention and treatment strategies. In clinical settings, assessing markers such as NU or targeting vmPFC‐related functions could assist in identifying individuals at higher risk for escalating to SUD, even if their substance use appears moderate.

## Author Contributions

MKH, RW and TE conceptualised the study. KS led the data analysis and drafted the original manuscript. MKH was responsible for data curation, methodology, investigation, project administration and contributed to visualisation. NS handled data acquisition and analysis. RW and TE secured funding and provided supervision. All authors contributed to manuscript review and editing.

## Ethics Statement

The authors assert that all procedures contributing to this work comply with the ethical standards of the relevant national and institutional committees on human experimentation and with the Helsinki Declaration of 1975, as revised in 2013. All procedures involving human subjects/patients were approved by the local Ethics Committee (Ethikkommission der Technischen Universität Dresden [institutional review board: IRB00001473]—approval number: SR‐EK‐270062020).

## Conflicts of Interest

The authors declare no conflicts of interest.

## Supporting information


**Table S1:** Sociodemographic and clinical characteristics of participants.
**Figure S1:** Number of substances consumed by the participants.
**Figure S2:** substance‐specific severity of SUD in the current sample.
**Figure S3:** Regions of interest (ROI) used in the current study.
**Table S2:** Anatomical regions showing significant negative associations between grey matter volume and substance‐related problems, the degree of use, and problems controlled for use in whole‐brain linear regression analysis.
**Table S3:** Regression results using substance‐related problems as the criterion and bilateral insula as the regressor of interest (*R* ^2^ = 0.130**).
**Table S4:** Regression results using substance‐related problems as the criterion and anterior cingulate cortex (ACC) as the regressor of interest (*R* ^2^ = 0.108**).
**Table S5:** Regression results using substance‐related problems as the criterion and medial prefrontal cortex (mPFC) as the regressor of interest (*R* ^2^ = 0.206**).
**Table S6:** Regression results using substance‐related problems at follow‐up as the criterion and insula as the regressor of interest (*R* ^2^ = 0.142*).
**Table S7:** Regression results using substance‐related problems at follow‐up as the criterion and anterior cingulate cortex (ACC) as the regressor of interest (*R* ^2^ = 0.108*).
Table S8: Regression results using substance‐related problems at follow‐up as the criterion and medial prefrontal cortex (mPFC) as the regressor of interest (*R* ^2^ = 0.172**).
**Table S9:** Regression results using degree of use as the criterion and bilateral insula as the regressor of interest (*R* ^2^ = 0.132**).
**Table S10:** Regression results using degree of use as the criterion and anterior cingulate cortex (ACC) as the regressor of interest (*R* ^2^ = 0.121**).
**Table S11:** Regression results using degree of use as the criterion and medial prefrontal cortex (mPFC) as the regressor of interest (*R* ^2^ = 0.215**).
**Table S12:** Regression results using degree of use at follow‐up as the criterion and bilateral insula as the regressor of interest (*R* ^2^ = 0.065).
**Table S13:** Regression results using degree of use at follow‐up as the criterion and anterior cingulate cortex (ACC) as the regressor of interest (*R* ^2^ = 0.064).
**Table S14:** Regression results using degree of use at follow‐up as the criterion and medial prefrontal cortex (mPFC) as the regressor of interest (*R* ^2^ = 0.124**).
**Table S15:** Regression results using substance‐related problems as the criterion and bilateral insula as the regressor of interest (*R* ^2^ = 0.495**).
**Table S16:** Regression results using substance‐related problems as the criterion and anterior cingulate cortex (ACC) as the regressor of interest (*R* ^2^ = 0.489**).
**Table S17:** Regression results using substance‐related problems as the criterion and medial prefrontal cortex (mPFC) as the regressor of interest (*R* ^2^ = 0.502**).
**Table S18:** Regression results using substance‐related problems at follow‐up as the criterion and bilateral insula as the regressor of interest (*R* ^2^ = 0.474**).
**Table S19:** Regression results using substance‐related problems at follow‐up as the criterion and anterior cingulate cortex (ACC) as the regressor of interest (*R* ^2^ = 0.454**).
**Table S20:** Regression results using substance‐related problems at follow‐up as the criterion and medial prefrontal cortex (mPFC) as the regressor of interest (*R* ^2^ = 0.466**).
**Table S21:** Regression results using NU as the criterion and bilateral insula as the regressor of interest (*R* ^2^ = 0.024).
**Table S22:** Regression results using NU as the criterion and anterior cingulate cortex (ACC) as the regressor of interest (*R* ^2^ = 0.048).
**Table S23:** Regression results using NU as the criterion and medial prefrontal cortex (mPFC) as the regressor of interest (*R* ^2^ = 0.072*).
**Table S24:** Partial correlation results between the predefined ROIs (i.e., ACC, bilateral insula and dorsal striatum) and substance‐related problems, degree of use and substance‐related problems controlled for use (*N* = 134).
**Table S25:** Partial correlation results between ROIs (i.e., ACC, bilateral insula and vmPFC) and substance‐related problems, degree of use and substance‐related problems controlled for use in the smaller follow‐up sample (*N* = 120).
**Table S26:** Correlation analyses between brain parameters (mean GMV in the ROIs [i.e., ACC, bilateral Insula, mPFC], TIV, demographic information and substance‐related measures [i.e., degree of use and substance‐related problems]).

## Data Availability

I, Kristina Schwarz, declare that the manuscript is an honest, accurate and transparent account of the study. No important aspects of the study have been omitted, and any discrepancies from the study as planned and preregistered in OSF (https://osf.io/9b35a) have been explained. The data and main analyses code that support the findings of this study can be accessed here: https://osf.io/bz75k/.
