# Supplementary material for: Grey Matter Volume in Substance Use: A Preregistered, Dimensional Approach to Disentangle Substance Use and Disorder Severity
Source: Addict Biol. 2025 Jul 28;30(8):e70075. doi: 10.1111/adb.70075 (PMC12302895; doi:10.1111/adb.70075)
Supplement: Supplementary file 1 — Table S1: Sociodemographic and clinical characteristics of participants. Figure S1: Number of substances consumed by the participants. Figure S2: substance‐specific severity of SUD in the current sample. Figure S3: Regions of interest (ROI) used in the current study. Table S2: Anatomical regions showing significant negative associations between grey matter volume and substance‐related problems, the degree of use, and problems controlled for use in whole‐brain linear regression analysis. Table S3: Regression results using substance‐related problems as the criterion and bilateral insula as the regressor of interest (R 2 = 0.130**). Table S4: Regression results using substance‐related problems as the criterion and anterior cingulate cortex (ACC) as the regressor of interest (R 2 = 0.108**). Table S5: Regression results using substance‐related problems as the criterion and medial prefrontal cortex (mPFC) as the regressor of interest (R 2 = 0.206**). Table S6: Regression results using substance‐related problems at follow‐up as the criterion and insula as the regressor of interest (R 2 = 0.142*). Table S7: Regression results using substance‐related problems at follow‐up as the criterion and anterior cingulate cortex (ACC) as the regressor of interest (R 2 = 0.108*). Table S8: Regression results using substance‐related problems at follow‐up as the criterion and medial prefrontal cortex (mPFC) as the regressor of interest (R 2 = 0.172**). Table S9: Regression results using degree of use as the criterion and bilateral insula as the regressor of interest (R 2 = 0.132**). Table S10: Regression results using degree of use as the criterion and anterior cingulate cortex (ACC) as the regressor of interest (R 2 = 0.121**). Table S11: Regression results using degree of use as the criterion and medial prefrontal cortex (mPFC) as the regressor of interest (R 2 = 0.215**). Table S12: Regression results using degree of use at follow‐up as the criterion and bilateral insula as the regres [file ADB-30-e70075-s001.pdf]

# Supplementary information

## Gray Matter Volume Differences in Substance Users - A Dimensional Approach to Disentangle Substance Use and Disorder

Schwarz & Hildebrandt, et al.

**Comment:** This paper has been preregistered on the Open Science Framework (https://osf.io/9b35a). We tried to strictly follow the preregistered approach. Adaptations made are highlighted in the following text and mainly concerns the definition of Regions of Interest that were adapted in order to facilitate the analyses, preserve statistical power and incorporate new studies that have been published since preregistration.

## 1 Methods

### 1.1 Sample Description

Participants were recruited through advertisements in nightclubs, bars and restaurants, social media channels affiliated with the party scene in Dresden and the city in general, and counseling centers. Some participants ($n$ = 41) were recruited from the sample of a precursory study.[1] The remaining subjects first participated in a brief online survey and a telephone interview assessing inclusion and exclusion criteria. For inclusion, participants needed to be current substance users (at least one psychoactive substance except for caffeine at least once per month in the preceding year), aged between 18 to 35 years, have sufficient understanding of the German language, be right-handed, free from contraindications to MRI scanning, and report their first use of any substance to be at least one year ago. Exclusion criteria included current (major depressive episode, suicidality, eating disorders, attention deficit hyperactivity disorder (ADHD)) and lifetime (schizophrenia, psychotic episode, manic episode, borderline personality disorder, intermittent explosive disorder) psychopathology as well as lifetime neurological disorders (migraine, brain injuries, severe head trauma with unconsciousness). After data collection, one participant was excluded due to an ADHD diagnosis that had not been detected before, and two were excluded due to brain anomalies (extremely large ventricles, incidental finding). The final sample included 134 participants (3 diverse, 53 female, 78 male). Detailed demographic and substance use characteristics of the sample are presented in Table 1 and Table S1. The participants were asked to abstain from using psychoactive substances for at least the five-fold of the respective plasma half-life of each substance (except for nicotine and caffeine ≥ 1 hour) prior to their appointment.[2] On the study day, breath alcohol was tested, and participants confirmed compliance with the abstinence periods. Only individuals without acute

withdrawal symptoms (assessed with the SCID-5 withdrawal section (German Version[3]) were permitted to participate.

**Table S1:** *Sociodemographic and clinical characteristics of participants*

| Characteristic | *N* | % |
|---|---|---|
| **Migration Background** | 19 | 14.2 |
| **Self-identified Ethnicity** | | |
| Asian/German-Asian | 1 | 0.7 |
| Indigenous American | 1 | 0.7 |
| Middle East / North African | 1 | 0.7 |
| Sinti*zze and Rom*nja | 2 | 1.5 |
| White | 125 | 93.3 |
| Preferred not to say | 4 | 3.0 |
| **DSM-5 Diagnoses** | | |
| *current* | | |
| Social Anxiety Disorder | 5 | 3.7 |
| Specific Phobia | 1 | 0.7 |
| Generalized Anxiety Disorder | 3 | 2.2 |
| Post-traumatic Stress Disorder | 2 | 1.5 |
| Pre-menstrual Dysphoric Disorder | 7 | 5.2 |
| Insomnia | 7 | 5.2 |
| Hypersomnia | 3 | 2.2 |
| Dysmorphophobia | 1 | 0.7 |
| Gambling Disorder | 1 | 0.7 |
| *lifetime* | | |
| Major Depression | 46 | 34.3 |
| Panic Disorder | 4 | 3.0 |
| Post-traumatic Stress Disorder | 11 | 8.2 |

*Note.* Migration background defined as one parent born abroad. All data were collected during the laboratory session. $N = 134$.

### 1.3 Operationalization and Calculation of substance scores

*Degree of Substance use:* The Degree of substance use and substance-related problems were assessed as previously reported [1,4]. In short, substance use was assessed with a detailed questionnaire including frequency and quantity (scale ranging from 0 (nothing) to 5 (very much)) for each substance used in the past year. The degree of substance use was operationalized as the total sum of the substance-specific use scores (frequency x subjective quantity). Data were ordered quantile transformed to ensure a normalized distribution. See Figure S1 for the number of substances consumed by the participants.

**Figure S1: Number of substances consumed by the participants**

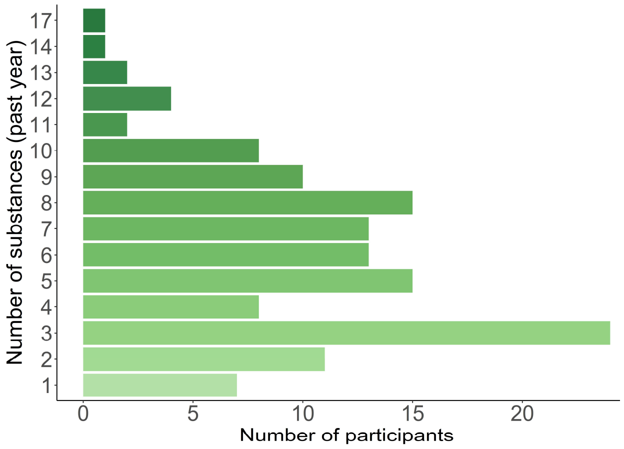


*Substance-related problems:* Substance-related problem were assessed by trained clinical psychologists (and re-evaluated by a second independent rater) using the SUD section of the SCID-5 interview[3] for each substance used within the past year, supplemented by severity ratings for each DSM-5 A-criterion symptom (scale ranging from 0 (not present) to 3 (extreme)). Each interview was checked for compliance with the stringent coding system and then re-evaluated by a second independent rater resulting in excellent interrater reliability (intraclass correlation .989). We used the sum of severity ratings across all substances to operationalize problems. See Figure S2 for substance-specific SUD severities in the current sample.

**Figure S2: substance-specific severity of SUD in the current sample**

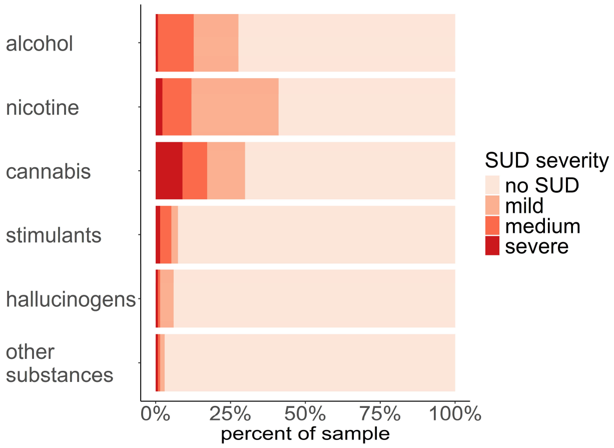


## 1.4 MRI data processing

Preprocessing in the Computational Anatomy Toolbox (CAT12[5] included noise removal, bias-correction, affine registration, and the initial segmentation of gray matter, white matter, and cerebrospinal fluid. Skull-stripping, brain parcellation and hyperintensity corrections were performed, followed by the final adaptive maximum a posteriori segmentation (AMAP;[6]) as well as by partial volume estimation to account for partial volume effects.[7] The native-space tissue segments were normalized to MNI space using the Diffeomorphic Anatomic Registration through Exponentiated Lie algebra algorithm (DARTEL;[8]) to provide precise spatial inter-subject registration. To preserve the absolute gray matter volume within each voxel, the images were modulated by the Jacobian determinants that were calculated during spatial normalization by the DARTEL algorithm. The total intracranial volume (TIV) was estimated to correct for brain size and volume in subsequent analyses.[9] Lastly, images were smoothed with a 6-mm full-width-half-maximum (FWHM) Gaussian kernel.

## 1.5 ROI definition

In our preregistration ROI selection was based on a meta-analysis on volumetric differences between SUD and controls[10]. The map comprised (1) a bilateral frontal region (ACC/mPFC), the (2) left anterior and left and right posterior insula (see Figure S3A).

However, we decided to use structurally defined masks using a well-established brain atlas (Individual Brain Atlases using Statistical Parametric Mapping Software (IBASPM; [11]) in order to improve interpretability and comparability across studies. Specifically, the meta-analytic insula ROIs covered only parts of the insula, which would have restricted our discussion to the specific masks not the structure itself. Similarly, the meta-analytic prefrontal

regions covered parts of the ACC extending into the mPFC, two regions linked to SUD but with distinct mechanistic and functional roles. In order to enable a more precise and anatomically grounded distinction between the two areas, we separated both regions using the anatomic brain atlas (see Figure S3B).

In addition, we originally preregistered the (structurally defined) dorsal striatum as a ROI given a number of studies reporting GMV alterations in SUD in this region[12–14]. However, alterations were found less consistently and mostly in substance-specific studies only, while the meta-analyses across substances (mostly published after preregistration, e.g. [10,15–17]) did not detect GMV alterations in the dorsal striatum (see Ersche and colleagues[18] for a discussion). In order to preserve statistical power and reduce the number of statistical tests, we therefor decided to exclude the dorsal striatum ROI. See Figure S3 for the final set of ROIs. For transparency, results with the originally defined ROIs are reported in the supplementary section 2.3.1.

**Figure S3:** Regions of interest (ROI) used in the current study

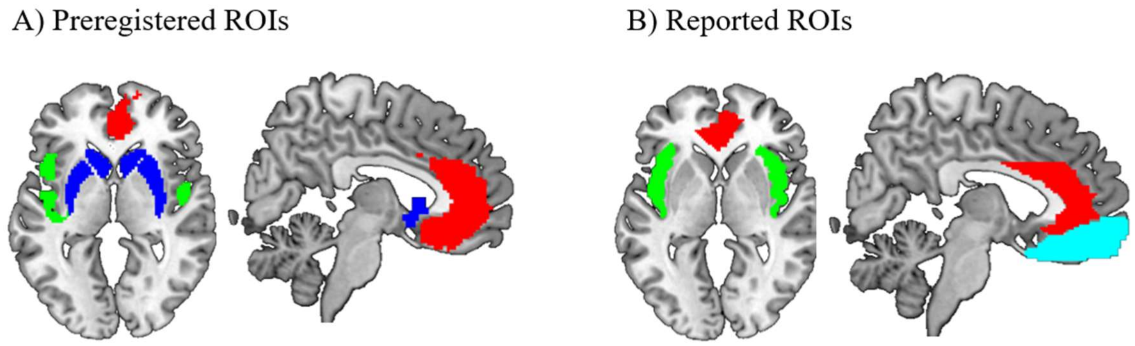


*Note*: structural images are derived from the Individual Brain Atlases using Statistical Parametric Mapping Software (IBASPM; [11]; atlas 116). Green: bilateral insula comprising of are 29 [Insula_L] and 30 [Insula_R]; cyan: mPFC comprising area 25 [Frontal_Mid_Orb_L], 26 [Frontal_Mid_Orb_R], 27 [Rectus_L], 28 [Rectus_R]; red: ACC comprising area 31 [Cingulum_Ant_L] and 32 [Cingulum_Ant_R].

## 1.6 Explorative analyses of most-problematic substance use

To show that the main findings of this study are not due to aggregating across substances, we repeated the regression analyses of GMV in the ROIs on 1) substance-related problems 2) the degree of substance use, and 2) substance-related problems controlled for the degree of substance use, using substance-related measures from the individually most problematic substance (i.e., highest substance-specific problem score).

# 2 Results

## 2.1 Whole brain analyses

**Table S2:** *Anatomical regions showing significant negative associations between gray matter volume and substance-related problems, the degree of use, and problems controlled for use in whole-brain linear regression analysis*

| Brain region (AAL3) | MNI coordinates | | | *TFCE* | *k* | $p_{FWE}$ |
|---|---|---|---|---|---|---|
| | *x* | *y* | *z* | | | |
| **Cross-sectional Results** | | | | | | |
| *A) Substance-related problems* | | | | | | |
| L Gyrus rectus [Frontal medial cortex] | -3 | 40 | -15 | 1847.80 | 8849 | .001 |
| L Mid cingulate gyrus [Posterior cingulate gyrus] | -4 | -30 | 45 | 1133.51 | 545 | .023 |
| R Postcentral gyrus [Postcentral gyrus] | 30 | -30 | 56 | 1113.42 | 882 | .024 |
| L Mid cingulate gyrus [Juxtapositional lobule] | -8 | 6 | 45 | 1118.98 | 642 | .025 |
| L Mid frontal gyrus [Superior frontal gyrus] | -26 | 0 | 63 | 1090.77 | 306 | .028 |
| R [Frontal orbital cortex] | 28 | 27 | -4 | 1061.39 | 154 | .032 |
| L Anterior Cingulate gyrus [Anterior Cingulate Gyrus] | -9 | 21 | 27 | 1035.17 | 218 | .037 |
| *B) Degree of substance-use* | | | | | | |
| R Gyrus rectus [Frontal medial cortex] | 4 | 38 | -22 | 1718.00 | 5297 | .001 |
| L Supplementary motor area [Superior frontal gyrus] | -4 | 9 | 58 | 1101.70 | 223 | .027 |
| L Superior frontal gyrus [Superior frontal gyrus] | -20 | 4 | 63 | 1093.05 | 240 | .029 |
| R Olfactory gyrus [Subcallosal cortex] | 8 | 18 | -12 | 981.25 | 32 | .048 |
| *C) Substance-related problems beyond Degree of substance-use* | | | | | | |
| No results | | | | | | |

**Table S2 (continued):** *Anatomical regions showing significant negative associations between gray matter volume and substance-related problems, the degree of use, and problems controlled for use in whole-brain linear regression analysis*

| Brain region (AAL3) | MNI coordinates | | | *TFCE* | *k* | $p_{FWE}$ |
|---|---|---|---|---|---|---|
| | *x* | *y* | *z* | | | |
| **Follow-up Results** | | | | | | |
| *A) Substance-related problems* | | | | | | |
| L Supramarginal gyrus [Supramerginal gyrus] | -57 | -39 | 33 | 1137.01 | 473 | .024 |
| L postcentral gyrus [Postcentral gyrus] | -32 | -32 | 62 | 1038.34 | 58 | .038 |
| L precunes [Precuneus Cortex] | -9 | -51 | 56 | 999.21 | 17 | .045 |
| L postcentral gyrus [Postcentral gyrus] | -42 | -28 | 57 | 987.02 | 25 | .048 |
| *B) Degree of substance-use* | | | | | | |
| No results | | | | | | |
| *C) Substance-related problems beyond Degree of substance-use* | | | | | | |
| No results | | | | | | |

*Note*: Regions (*k* > 10 voxels) were classified according to the Automated Anatomical Labeling Atlas [19]complemented by functional labels of the Anatomical Probability Maps (Anatomy toolbox) in square brackets [20]. Cluster extent *k* is given at *P*corr < .05, familywise error (FWE) corrected for multiple comparisons using nonparametric Threshold-free Cluster Enhancement (TFCE) within the whole brain. *x*-, *y*-, and *z*-coordinates (MNI) and statistical information refer to the peak voxel(s) in the corresponding cluster (voxel-level statistics). R, right; L, left.

## 2.2 ROI analyses

### 2.2.1 Association between GMV and substance-related problems

**Cross-sectional Results**

**Table S3:** *Regression results using substance-related problems as the criterion and* *bilateral insula* *as the regressor of interest* ($R^2$ = .130**)

| Term | B | SE(B) | β | t | p | Tolerance | VIF | CI (95%) |
|---|---|---|---|---|---|---|---|---|
| (Intercept) | 2.014 | 1.030 | - | 1.956 | .053 | - | - | [-.023, 4.05 ] |
| TIV | .001 | .001 | .205 | 1.363 | .175 | .300 | 3.342 | [-.001, .003] |
| age | -.050* | .02 | -.228** | -2.574 | .011 | .860 | 1.163 | [-.09, -.01] |
| sex | .677** | .21 | .341** | 3.196 | .002 | .592 | 1.689 | [.26, 1.10] |
| **Insula** | **-.228*** | **.076** | **-.424*** | **-2.983** | **.002**[a] | **.334** | **3.00** | **[-.38, -.08]** |

*Note. β* indicates standardized regression weights. SE = standard error, VIF: Variance Inflation Factor, CI: confidence interval, TIV: Total Intracranial Volume, Boldface – regressor of interest. * indicates $p < .05$. ** indicates $p < .01$. [a] one-tailed.

**Table S4:** *Regression results using substance-related problems as the criterion and* *anterior cingulate cortex (ACC)* *as the regressor of interest* ($R^2$ = .108**)

| Term | B | SE(B) | β | t | p | Tolerance | VIF | CI (95%) |
|---|---|---|---|---|---|---|---|---|
| (Intercept) | 1.525 | 1.008 | - | 1.513 | .133 | - | - | [-.47, 3.52] |
| TIV | .001 | .001 | .116 | .803 | .423 | .332 | 3.014 | [-.001, .003] |
| age | -.048* | .020 | -.221* | -2.421 | .017 | .830 | 1.205 | [-.09, -.01] |
| sex | .512* | .215 | .258* | 2.375 | .019 | .587 | 1.705 | [.09, .94] |
| **ACC** | **-.219*** | **.093** | **-.291*** | **-2.368** | **.010** [a] | **.458** | **2.183** | **[-.40, -.04]** |

*Note.* β indicates standardized regression weights. SE = standard error, VIF: Variance Inflation Factor, CI: confidence interval, TIV: Total Intracranial Volume, Boldface – regressor of interest. * indicates p < .05. ** indicates p < .01. [a] one-tailed.

**Table S5:** *Regression results using substance-related problems as the criterion and* *medial prefrontal cortex (mPFC)* *as the regressor of interest* ($R^2$ = .206**)

| Term | B | SE(B) | β | t | p | Tolerance | VIF | CI (95%) |
|---|---|---|---|---|---|---|---|---|
| (Intercept) | 1.375 | .930 | - | 1.479 | .142 | - | - | [-.47, 3.21] |
| TIV | .003** | .001 | .465** | 2.921 | .004 | .243 | 4.113 | [-.001, .005] |
| age | -.065** | .019 | -.299** | -3.453 | <.001 | .820 | 1.219 | [-.10, -.03] |
| sex | .470* | .202 | .237* | 2.320 | .022 | .592 | 1.689 | [.07, .87] |
| **mPFC** | **-.433**** | **.092** | **-.660**** | **-4.698** | **<.001** | **.312** | **3.200** | **[-.62, -.25]** |

*Note.* β indicates standardized regression weights. SE = standard error, VIF: Variance Inflation Factor, CI: confidence interval, TIV: Total Intracranial Volume, Boldface – regressor of interest. * indicates p < .05. ** indicates p < .01.

## Follow-up Results

**Table S6:** *Regression results using substance-related problems at follow-up as the criterion and <u>insula</u> as the regressor of interest* ($R^2$ = .142*)

| Term | B | SE(B) | β | t | p | Tolerance | VIF | CI (95%) |
|---|---|---|---|---|---|---|---|---|
| (Intercept) | .937 | 1.077 | - | .870 | .386 | - | - | [-1.20, 3.07] |
| TIV | .001 | .001 | .295 | 1.898 | .060 | .309 | 3.241 | [.00, .004] |
| age | -.066** | .020 | -.297** | -3.210 | .002 | .873 | 1.145 | [-.12, -.03] |
| sex | .512* | .216 | .263* | 2.363 | .020 | .604 | 1.655 | [.08, .94] |
| **Insula** | **-.179*** | **.078** | **-.337*** | **-2.307** | **.012** [a] | **.350** | **2.854** | **[-.33, -.03]** |

*Note.* *β* indicates standardized regression weights. SE = standard error, VIF: Variance Inflation Factor, CI: confidence interval, TIV: Total Intracranial Volume, Boldface – regressor of interest. * indicates $p < .05$. ** indicates $p < .01$. [a] one-tailed.

**Table S7:** *Regression results using substance-related problems at follow-up as the criterion and <u>anterior cingulate cortex (ACC)</u> as the regressor of interest* ($R^2$ = .108*)

| Term | B | SE(B) | β | t | p | Tolerance | VIF | CI (95%) |
|---|---|---|---|---|---|---|---|---|
| (Intercept) | .311 | 1.064 | - | .293 | .770 | - | - | [-1.80, 2.42] |
| TIV | .001 | .001 | .128 | .841 | .402 | .333 | 3.004 | [-.001, .003] |
| age | -.058** | .022 | -.260** | -2.672 | .009 | .817 | 1.224 | [-.10, -.02] |
| sex | .425 | .223 | .218 | 1.908 | .059 | .593 | 1.686 | [-.02, .87] |
| **ACC** | **-.080** | **.096** | **-.109** | **-.837** | **.404** | **.454** | **2.204** | **[-.27, .11]** |

*Note.* *β* indicates standardized regression weights. SE = standard error, VIF: Variance Inflation Factor, CI: confidence interval, TIV: Total Intracranial Volume, Boldface – regressor of interest. * indicates $p < .05$. ** indicates $p < .01$.

**Table S8:** *Regression results using substance-related problems at follow-up as the criterion and <u>medial prefrontal cortex (mPFC)</u> as the regressor of interest* ($R^2$ = .172**)

| Term | B | SE(B) | β | t | p | Tolerance | VIF | CI (95%) |
|---|---|---|---|---|---|---|---|---|
| (Intercept) | .441 | 1.002 | - | .440 | .661 | - | - | [-1.54, 2.43] |
| TIV | .003** | .001 | .453** | 2.652 | .009 | .246 | 4.059 | [.001, .005] |
| age | -.076** | .021 | -.342** | -3.661 | <.001 | .824 | 1.214 | [-.12, -.04] |
| sex | .374 | .213 | .192 | 1.755 | .082 | .602 | 1.662 | [-.05, .80] |
| **mPFC** | **-.308**** | **.099** | **-.471**** | **-3.109** | **.001** [a] | **.313** | **3.192** | **[-.50, -.11]** |

*Note.* *β* indicates standardized regression weights. SE = standard error, VIF: Variance Inflation Factor, CI: confidence interval  TIV: Total Intracranial Volume, Boldface – regressor of interest. * indicates $p < .05$. ** indicates $p < .01$. [a] one-tailed.

### 2.2.2 Association between GMV and the degree of use

**Cross-Sectional Results**

**Table S9:** *Regression results using degree of use as the criterion and* bilateral insula *as the regressor of interest ($R^2$ = .132**)*

| Term | B | SE(B) | β | t | p | Tolerance | VIF | CI (95%) |
|---|---|---|---|---|---|---|---|---|
| (Intercept) | 3.406 | 1.051 | - | 3.243 | .002 | - | - | [1.33, 5.49] |
| TIV | .000 | .001 | .002 | .016 | .987 | .299 | 3.342 | [-.002, .002] |
| age | -.063** | .020 | -.284** | -3.208 | .002 | .860 | 1.163 | [-.10, -.02] |
| sex | .693** | .216 | .342** | 3.209 | .002 | .592 | 1.689 | [.23, 1.12] |
| **Insula** | **-.174*** | **.078** | **-.317*** | **-2.236** | **.014[a]** | **.334** | **2.995** | **[-.33, -.02]** |

*Note. β* indicates standardized regression weights. SE = standard error, VIF: Variance Inflation Factor, CI: confidence interval, TIV: Total Intracranial Volume, Boldface – regressor of interest. * indicates $p < .05$. ** indicates $p < .01$. [a] one-tailed.

**Table S10:** *Regression results using degree of use as the criterion and* anterior cingulate cortex (ACC) *as the regressor of interest ($R^2$ = .121**)*

| Term | B | SE(B) | β | t | p | Tolerance | VIF | CI (95%) |
|---|---|---|---|---|---|---|---|---|
| (Intercept) | 3.040 | 1.002 | - | 2.973 | .004 | - | - | [1.02, 5.06] |
| TIV | .000 | .001 | -.061 | -.426 | .671 | .332 | 3.014 | [-.002, .001] |
| age | -.062** | .020 | -.279** | -3.085 | .002 | .830 | 1.205 | [-.10, -.02] |
| sex | .566* | .218 | .279* | 2.589 | .011 | .587 | 1.705 | [.13, 1.00] |
| **ACC** | **-.171*** | **.094** | **-.222*** | **-1.817** | **.036[a]** | **.458** | **2.183** | **[-.36, -.02]** |

*Note.* β indicates standardized regression weights. SE = standard error, VIF: Variance Inflation Factor, CI: confidence interval, TIV: Total Intracranial Volume, Boldface – regressor of interest. * indicates p < .05. ** indicates p < .01. [a] one-tailed.

**Table S11:** *Regression results using degree of use as the criterion and* medial prefrontal cortex (mPFC) *as the regressor of interest ($R^2$ = .215**)*

| Term | B | SE(B) | β | t | p | Tolerance | VIF | CI (95%) |
|---|---|---|---|---|---|---|---|---|
| (Intercept) | 2.990 | .944 | - | 3.166 | .002 | - | - | [1.12, 4.86] |
| TIV | .002 | .001 | .299 | 1.888 | .061 | .243 | 4.113 | [.00, .004] |
| age | -.081** | .019 | -.365** | -4.239 | <.001 | .820 | 1.219 | [-.12, -.04] |
| sex | .512* | .206 | .253* | 2.493 | .014 | .592 | 1.689 | [.11, .92] |
| **mPFC** | **-.408**** | **.094** | **-.609**** | **-4.366** | **<.001** | **.312** | **3.200** | **[-.59, -.22]** |

*Note.* β indicates standardized regression weights. SE = standard error, VIF: Variance Inflation Factor, CI: confidence interval, TIV: Total Intracranial Volume, Boldface – regressor of interest. * indicates p < .05. ** indicates p < .01.

## Follow-up Results

**Table S12:** *Regression results using degree of use at follow-up as the criterion and bilateral insula as the regressor of interest ($R^2$ = .065)*

| Term | B | SE(B) | β | t | p | Tolerance | VIF | CI (95%) |
|---|---|---|---|---|---|---|---|---|
| (Intercept) | .311 | 1.165 | - | .266 | .790 | - | - | [-2.00, 2.62] |
| TIV | .001 | .001 | .145 | .893 | .374 | .309 | 3.241 | [-.001, .003] |
| age | -.038 | .022 | -.166 | -1.724 | .087 | .873 | 1.145 | [-.08, .01] |
| sex | .435 | .234 | .215 | 1.856 | .066 | .604 | 1.655 | [-.03, .90] |
| **Insula** | **-.091** | **.084** | **-.166** | **-1.088** | **.279** | **.350** | **2.854** | **[-.26, .08]** |

*Note.* *β* indicates standardized regression weights. SE = standard error, VIF: Variance Inflation Factor, CI: confidence interval, TIV: Total Intracranial Volume, Boldface – regressor of interest. * indicates $p < .05$. ** indicates $p < .01$.

**Table S13:** *Regression results using degree of use at follow-up as the criterion and anterior cingulate cortex (ACC) as the regressor of interest ($R^2$ = .064)*

| Term | B | SE(B) | β | t | p | Tolerance | VIF | CI (95%) |
|---|---|---|---|---|---|---|---|---|
| (Intercept) | .156 | 1.130 | - | .138 | .890 | - | - | [-2.08, 2.40] |
| TIV | .001 | .001 | .129 | .822 | .413 | .333 | 3.004 | [-.001, .003] |
| age | -.040 | .023 | -.173 | -1.738 | .085 | .817 | 1.224 | [-.19, .01] |
| sex | .366 | .237 | .181 | 1.545 | .125 | .593 | 1.686 | [-.10, .83] |
| **ACC** | **-.104** | **.102** | **-.137** | **-1.022** | **.309** | **.454** | **2.204** | **[-.31, .10]** |

*Note.* *β* indicates standardized regression weights. SE = standard error, VIF: Variance Inflation Factor, CI: confidence interval, TIV: Total Intracranial Volume, Boldface – regressor of interest. * indicates $p < .05$. ** indicates $p < .01$.

**Table S14:** *Regression results using degree of use at follow-up as the criterion and medial prefrontal cortex (mPFC) as the regressor of interest ($R^2$ = .124**)*

| Term | B | SE(B) | β | t | p | Tolerance | VIF | CI (95%) |
|---|---|---|---|---|---|---|---|---|
| (Intercept) | .233 | 1.069 | - | .218 | .828 | - | - | [-1.88, 2.35] |
| TIV | .003* | .001 | .429* | 2.438 | .016 | .246 | 4.059 | [.001, .005] |
| age | -.057* | .022 | -.246* | -2.561 | .012 | .824 | 1.214 | [-.10, -.01] |
| sex | .322 | .227 | .159 | 1.415 | .160 | .602 | 1.662 | [-.13, .77] |
| **mPFC** | **-.316**** | **.106** | **-.468**** | **-2.999** | **.002** [a] | **.313** | **3.192** | **[-.53, -.12]** |

*Note.* *β* indicates standardized regression weights. SE = standard error, VIF: Variance Inflation Factor, CI: confidence interval, TIV: Total Intracranial Volume, Boldface – regressor of interest. * indicates $p < .05$. ** indicates $p < .01$. [a] one-tailed.

### 2.2.3 Association between GMV and substance-related problems controlled for the degree of use

**Cross-sectional Results**

**Table S15:** *Regression results using substance-related problems as the criterion and bilateral insula as the regressor of interest ($R^2$ = .495**)*

| Term | B | SE(B) | β | t | p | Tolerance | VIF | CI (95%) |
|---|---|---|---|---|---|---|---|---|
| (intercept) | -.150 | .819 | - | -.183 | .855 | - | - | [-1.77, 1.47] |
| TIV | .001 | .001 | .203 | 1.769 | .079 | .299 | 3.342 | [.00, .003] |
| age | -.010 | .015 | -.044 | -.624 | .534 | .797 | 1.255 | [-.04, .02] |
| sex | .237 | .168 | .119 | 1.406 | .162 | .548 | 1.824 | [-.10, .57] |
| degree of use | .635** | .066 | .649** | 9.626 | <.001 | .868 | 1.153 | [.51, .77] |
| **Insula** | **-.117*** | **.060** | **-.218*** | **-1.968** | **.025 [a]** | **.321** | **3.111** | **[-.24, .001]** |

*Note. β* indicates standardized regression weights. SE = standard error, VIF: Variance Inflation Factor, CI: confidence interval, TIV: Total Intracranial Volume, Boldface – regressor of interest. * indicates $p < .05$. ** indicates $p < .01$. [a] one-tailed.

**Table S16:** *Regression results using substance-related problems as the criterion and anterior cingulate cortex (ACC) as the regressor of interest ($R^2$ = .489**)*

| Term | B | SE(B) | β | t | p | Tolerance | VIF | CI (95%) |
|---|---|---|---|---|---|---|---|---|
| (Intercept) | -.434 | .792 | - | -.548 | .585 | - | - | [-2.00, 1.13] |
| TIV | .001 | .001 | .156 | 1.422 | .157 | .331 | 3.018 | [.00, .002] |
| age | -.008 | .016 | -.037 | -.515 | .608 | .773 | 1.294 | [-.04, .02] |
| sex | .147 | .168 | .074 | .876 | .383 | .558 | 1.794 | [-.19, .48] |
| degree of use | .644** | .066 | .658** | 9.779 | <.001 | .879 | 1.138 | [.51, .78] |
| **ACC** | **-.109*** | **.071** | **-.145*** | **-1.534** | **.064 [a]** | **.447** | **2.239** | **[-.25, .03]** |

*Note. β* indicates standardized regression weights. SE = standard error, VIF: Variance Inflation Factor, CI: confidence interval, TIV: Total Intracranial Volume, Boldface – regressor of interest. * indicates $p < .05$. ** indicates $p < .01$.

**Table S17:** *Regression results using substance-related problems as the criterion and medial prefrontal cortex (mPFC) as the regressor of interest ($R^2$ = .502**)*

| Term | B | SE(B) | β | t | p | Tolerance | VIF | CI (95%) |
|---|---|---|---|---|---|---|---|---|
| (Intercept) | -.423 | .767 | - | -.552 | .450 | - | - | [-1.94, 1.09] |
| TIV | .002* | .001 | .281* | 2.194 | .015 | .237 | 4.227 | [.00, .003] |
| age | -.016 | .016 | -.075 | -1.018 | .248 | .720 | 1.389 | [-.05, .02] |
| sex | .161 | .165 | .081 | .980 | .524 | .565 | 1.770 | [-.16, .49] |
| degree of use | .601** | .069 | .614** | 8.729 | <.001 | .785 | 1.274 | [.47, .74] |
| **mPFC** | **-.187**** | **.078** | **-.285**** | **-2.385** | **.010 [a]** | **.272** | **3.673** | **[-.34, -.03]** |

*Note. β* indicates standardized regression weights. SE = standard error, VIF: Variance Inflation Factor, CI: confidence interval, TIV: Total Intracranial Volume, Boldface – regressor of interest. * indicates $p < .05$. ** indicates $p < .01$. [a] one-tailed.

## Follow-up Results

**Table S18:** *Regression results using substance-related problems at follow-up as the criterion and* *bilateral insula* *as the regressor of interest* ($R^2$ = .474**)

| Term | B | SE(B) | β | t | p | Tolerance | VIF | CI (95%) |
|---|---|---|---|---|---|---|---|---|
| (Intercept) | .759 | .847 | - | .895 | .372 | - | - | [-.92, 2.44] |
| TIV | .001 | .001 | .209 | 1.701 | .092 | .306 | 3.263 | [.000, .003] |
| age | -.044 | .016 | -.198 | -2.683 | .008 | .851 | 1.175 | [-.08, -.01] |
| sex | .262 | .173 | .134 | 1.514 | .133 | .586 | 1.705 | [-.08, .60] |
| degree of use | .574 | .068 | .595 | 8.474 | <.001 | .935 | 1.069 | [.44, .71] |
| **Insula** | **-.127** | **.061** | **-.238** | **-2.062** | **.021** [a] | **.347** | **2.883** | **[-.25, -.01]** |

*Note.* *β* indicates standardized regression weights. SE = standard error, VIF: Variance Inflation Factor, CI: confidence interval, TIV: Total Intracranial Volume, Boldface – regressor of interest. * indicates $p < .05$. ** indicates $p < .01$. [a] one-tailed.

**Table S19:** *Regression results using substance-related problems at follow-up as the criterion and* *anterior cingulate cortex (ACC)* *as the regressor of interest* ($R^2$ = .454**)

| Term | B | SE(B) | β | t | p | Tolerance | VIF | CI (95%) |
|---|---|---|---|---|---|---|---|---|
| (Intercept) | .220 | .836 | - | .263 | .793 | - | - | [-1.44, 1.88] |
| TIV | .000 | .001 | .050 | .417 | .678 | .331 | 3.021 | [-.001, .002] |
| age | -.034 | .017 | -.155 | -1.997 | .048 | .796 | 1.256 | [-.07, .000] |
| sex | .211 | .177 | .108 | 1.192 | .236 | .581 | 1.721 | [-.14, .56] |
| degree of use | .587 | .069 | .608 | 8.508 | <.001 | .936 | 1.068 | [.45, .72] |
| **ACC** | **-.019** | **.076** | **-.026** | **-.254** | **.800** | **.450** | **2.224** | **[-.17, .13]** |

*Note.* *β* indicates standardized regression weights. SE = standard error, VIF: Variance Inflation Factor, CI: confidence interval, TIV: Total Intracranial Volume, Boldface – regressor of interest. * indicates $p < .05$. ** indicates $p < .01$.

**Table S20:** *Regression results using substance-related problems at follow-up as the criterion and* *medial prefrontal cortex (mPFC)* *as the regressor of interest* ($R^2$ = .466**)

| Term | B | SE(B) | β | t | p | Tolerance | VIF | CI (95%) |
|---|---|---|---|---|---|---|---|---|
| (Intercept) | .311 | .809 | - | .384 | .701 | - | - | [-1.29, 1.91] |
| TIV | .001 | .001 | .205 | 1.451 | .150 | .234 | 4.268 | [.00, .003] |
| age | -.044* | .017 | -.200* | -2.575 | .011 | .779 | 1.283 | [-.08, -.01] |
| sex | .194 | .173 | .100 | 1.121 | .265 | .591 | 1.691 | [-.15, .54] |
| degree of use | .558** | .071 | .579** | 7.916 | <.001 | .876 | 1.141 | [.42, .70] |
| **mPFC** | **-.131** | **.083** | **-.201** | **-1.579** | **.117** | **.291** | **3.441** | **[-.30, .03]** |

*Note.* *β* indicates standardized regression weights. SE = standard error, VIF: Variance Inflation Factor, CI: confidence interval, TIV: Total Intracranial Volume, Boldface – regressor of interest. * indicates $p < .05$. ** indicates $p < .01$.

## 2.2.4 NU results

### 2.2.4.1 Association between GMV and NU

**Table S21:** *Regression results using NU as the criterion and* *bilateral insula* *as the regressor of interest* ($R^2$ = .024)

| Term | B | SE(B) | β | t | p | Tolerance | VIF | CI (95%) |
|---|---|---|---|---|---|---|---|---|
| (Intercept) | 28.769 | 7.658 | - | 3.757 | <.001 | - | - | [13.62, 43.92] |
| TIV | .005 | .007 | .111 | .698 | .487 | .299 | 3.342 | [-.01, .02] |
| age | -.224 | .144 | -.146 | -1.556 | .122 | .860 | 1.163 | [-.51, .06] |
| sex | -.955 | 1.575 | -.068 | -.606 | .546 | .592 | 1.689 | [-4.07, 2.16] |
| **Insula** | **-.155** | **.569** | **-.041** | **-.273** | **.785** | **.334** | **2.995** | **[-1.28, .97]** |

*Note. β* indicates standardized regression weights. SE = standard error, VIF: Variance Inflation Factor, CI: confidence interval, TIV: Total Intracranial Volume, Boldface – regressor of interest. * indicates $p < .05$. ** indicates $p < .01$. [a] one-tailed.

**Table S22:** *Regression results using NU as the criterion and* *anterior cingulate cortex (ACC)* *as the regressor of interest* ($R^2$ = .048)

| Term | B | SE(B) | β | t | p | Tolerance | VIF | CI (95%) |
|---|---|---|---|---|---|---|---|---|
| (Intercept) | 31.124 | 7.315 | - | 4.255 | <.001 | - | - | [16.65, 45.60] |
| TIV | .0112 | .007 | .262 | 1.756 | .081 | .332 | 3.014 | [-.001, .03] |
| age | -.310* | .145 | -.202* | -2.147 | .034 | .830 | 1.205 | [-.60, -.02] |
| sex | -1.470 | 1.563 | -.105 | -.940 | .349 | .587 | 1.705 | [-4.56, 1.62] |
| **ACC** | **-1.216** | **.627** | **-.230** | **-1.809** | **.073** | **.458** | **2.183** | **[-2.55, .114]** |

*Note. β* indicates standardized regression weights. SE = standard error, VIF: Variance Inflation Factor, CI: confidence interval, TIV: Total Intracranial Volume, Boldface – regressor of interest. * indicates $p < .05$. ** indicates $p < .01$.

**Table S23:** *Regression results using NU as the criterion and* *medial prefrontal cortex (mPFC)* *as the regressor of interest* ($R^2$ = .072*)

| Term | B | SE(B) | β | t | p | Tolerance | VIF | CI (95%) |
|---|---|---|---|---|---|---|---|---|
| (Intercept) | 29.745 | 7.056 | - | 4.216 | <.001 | - | - | [15.79, 43.70] |
| TIV | .019* | .008 | .425* | 2.472 | .015 | .248 | 4.113 | [.004, .04] |
| age | -.357* | .144 | -.233* | -2.489 | .014 | .820 | 1.219 | [-.64, -.07] |
| sex | -1.533 | 1.536 | -.110 | -.998 | .320 | .592 | 1.6889 | [-4.57, 1.51] |
| **mPFC** | **-1.810*** | **.699** | **-.393*** | **-2.590** | **.011** | **.312** | **3.200** | **[-3.19, -.43]** |

*Note. β* indicates standardized regression weights. SE = standard error, VIF: Variance Inflation Factor, CI: confidence interval, TIV: Total Intracranial Volume, Boldface – regressor of interest. * indicates $p < .05$. ** indicates $p < .01$.

### 2.2.4.2 Mediation analysis

A mediation analysis was conducted to examine whether negative urgency (NU) mediates the relationship between GMV in the mPFC) and substance-related problems (controlled for the degree of use) (see Fig. 3C)**.**

1. Significant **Total Effect** (X (mPFC volume) → Y (problems controlled for use)): $b$ = −0.22, $SE$ =.016, $T$ = −2.19, $p$ =.030, 95% $CI$ [−.42,−.02].
2. Significant **Path a** (X (mPFC volume) → M (NU)): $b$ = −1.8, $SE$ = .70, $T$ = −2.59, $p$ = .011, 95% $CI$ [−3.19, −.43].
3. Significant **Path b** (M (NU) → Y (problems controlled for use)): $b$ = .042, $SE$ = .012, $T$ = 3.42, $p$ = .001, 95% $CI$ [.02, .07].
4. Nonsignificant **Direct Effect** (X (mPFC volume) → Y (problems controlled for use), controlling for M (NU)): $b$ = −.15, $SE$ =.10, $T$ = −1.46, $p$ = .146 95% $CI$ [−.35, .05].
5. Significant **indirect Effect** (X (mPFC volume) → M (NU) → Y (problems controlled for use)), as indicated by the bootstrap confidence interval, $b$ = −.076, BootSE = .038, 95% $CI$ [−.12, −.02].

These results suggest that the relationship between mPFC volume and substance-related problems (controlled for use) is fully mediated by negative urgency**,** as the direct effect becomes non-significant after accounting for the mediator.

## 2.3 Additional explorative analyses

### 2.3.1 Partial Correlation analyses in originally preregistered ROIs

**Table S24:** *Partial Correlation Results between the predefined ROIs (i.e., ACC, bilateral Insula, dorsal striatum) and substance-related problems, degree of use and substance-related problems controlled for use (N=134)*

| | | ACC | bilateral insula | Dorsal striatum |
|---|---|---|---|---|
| Substance-Related Problems | r | -.276** | -.193* | .015 |
| | p-value | .001 | .027 | 0.864 |
| Degree of Use | r | -.222* | -.092 | -.047 |
| | p-value | .011 | .294 | .593 |
| Substance-Related Problems | r | -.175* | -.178* | .063 |
| (controlled for Use) | p-value | .046 | .042 | .473 |

Note: age, sex, Total Intracranial Volume were included as covariates. * indicates $p < .05$. ** indicates $p < .01$.

Results in the predefined ROIs are largely consistent with the reported ROIs showing significant negative associations between: 1) substance-related problems and ACC and insula (p < .027) but not dorsal striatal volume (p > .864); 2) the degree of substance use and ACC (p = .011) but not insula or dorsal striatal volume (p > .294); and 3) substance-related problems controlled for the degree of use and ACC and insula (p <. 046) but not dorsal striatal volume (p=.483).

### 2.3.2 Partial Correlation analyses in follow-up sample

**Table S25:** *Partial Correlation Results between ROIs (i.e., ACC, bilateral Insula, vmPFC) and substance-related problems, degree of use and substance-related problems controlled for use in the smaller follow-up sample (N=120)*

| | | ACC | Insula | mPFC |
|---|---|---|---|---|
| Substance-Related Problems | r | -.171* | -.241** | -.374* |
| | p-value | .032 | .005 | <.001 |
| Degree of Use | r | -.148 | -.188* | -.360** |
| | p-value | .055 | .020 | <.001 |
| Substance-Related Problems | r | -.098 | -.159* | -.192* |
| (controlled for Use) | p-value | .149 | .045 | .02 |

Note: age, sex, Total Intracranial Volume were included as covariates. * indicates $p < .05$. ** indicates $p < .01$.

In order to address the question of selective attrition that might have lead more severely affected individuals to drop out after the baseline assessment, baseline analyses were repeated in the subsample of individuals that completed both baseline and follow-up assessments. Results are consistent with the full sample, making it unlikely that selective attrition explains the non-significant problem-specific association at follow-up.

### 2.3.3 Explorative analyses of most-problematic substance

*Substance-related problems:* We detected significant negative associations between GMV and substance-related problems in all ROIs (ACC: $p$ = .013, $\beta$ = -.31; insula: $p$ = .005, $\beta$ = -.42; vmPFC: $p$ = <.001, $\beta$ = -.65).
*Degree of use:* We detected significant negative associations between GMV in the insula, vmPFC but not in the ACC and the degree of substance use (insula: $p$ = .037, $\beta$ = -.31; vmPFC: $p$ = .004, $\beta$ = -.44; ACC: $p$ = .203, $\beta$ = -.16).
*Substance-related problems controlled for the degree of use:* We detected significant negative associations between GMV and substance-related problems controlled for the degree of substance use in all ROIs (ACC: $p$ = .034, $\beta$ = -.47; insula: $p$ = .038, $\beta$ = -.27; vmPFC: $p$ < .001, $\beta$ = -.46).

### 2.3.4 Explorative analyses investigating the effect of lifetime MDD

*Substance-related problems:* Significant negative associations between GMV and substance-related problems were observed in all ROIs at baseline (ACC: $p_{BH}$ = .017, $\beta$ = -.28; insula: $p_{BH}$ = .001, $\beta$ = -.46; vmPFC: $p_{BH}$ = <.001, $\beta$ = -.61). At follow-up, problems were related to GMV in the insula and vmPFC (insula: $p_{BH}$ = .013, $\beta$ = -.36; vmPFC: $p_{BH}$ = .006, $\beta$ = -.44), but not the ACC ($p_{BH}$ = .189, $\beta$ = -.11)
*Degree of use:* GMV in all ROIs was negatively associated with the degree of substance use at baseline (insula: $p_{BH}$ = .026, $\beta$ = -.32; vmPFC: $p_{BH}$ = -.001, $\beta$ = -.59; ACC: $p_{BH}$ = .026, $\beta$ = -.45). At follow-up, only vmPFC volume remained associated with the degree of use ($p_{BH}$ = .006, $\beta$ = -.32), while no associations emerged for the insula (insula: $p_{BH}$ = .120, $\beta$ = -.18; ACC: $p_{BH}$ = .220, $\beta$ = -.16).
*Substance-related problems controlled for the degree of use:* At baseline, GMV in the insula and vmPFC was negatively associated with substance-related problems when controlling for use (insula: $p_{BH}$ = .029, $\beta$ = -.26; vmPFC: $p_{BH}$ = .031, $\beta$ = -.26) but not in the ACC ($p_{BH}$ = .092, $\beta$ = -.13). At follow-up, only the insula showed a significant problem-specific association ($p_{BH}$ = .042, $\beta$ = -.26), while vmPFC and ACC did not (vmPFC: $p_{BH}$ = .130, $\beta$ = -.20; ACC: $p_{BH}$ = .425, $\beta$ = -.02).
Including lifetime MDD diagnosis as an additional covariate did not change the overall pattern of results. The only difference was that the follow-up association in the insula reached significance when controlling for MDD ($p_{\text{withoutMDD}}$ = .063; $p_{\text{withMDD}}$ = .042).

### 2.3.3 Additional correlation analyses

**Table S27:** *Correlation Analyses between brain parameters (mean GMV in the ROIs (i.e., ACC, bilateral Insula, mPFC), TIV, demographic information and substance-related measures (i.e. degree of use and substance-related problems))*

| | | ACC | bilateral insula | mPFC | Substance-Related Problems | Degree of Use | Substance-Related Problems (T2) | Degree of Use (T2) | TIV | age |
|---|---|---|---|---|---|---|---|---|---|---|
| ACC | r | 1 | | | | | | | | |
| | p-value | | | | | | | | | |
| | N | 134 | | | | | | | | |
| bilateral insula | r | .710** | 1 | | | | | | | |
| | p-value | <0.001 | | | | | | | | |
| | N | 134 | 134 | | | | | | | |
| mPFC | r | .744** | .854** | 1 | | | | | | |
| | p-value | <0.001 | <0.001 | | | | | | | |
| | N | 134 | 134 | 134 | | | | | | |
| Substance-Related Problems | r | -0.090 | -0.058 | -0.152 | 1 | | | | | |
| | p-value | 0.302 | 0.505 | 0.079 | | | | | | |
| | N | 134 | 134 | 134 | 134 | | | | | |
| Degree of Use | r | -0.120 | -0.105 | -.216* | .681** | 1 | | | | |
| | p-value | 0.168 | 0.228 | 0.012 | <0.001 | | | | | |
| | N | 134 | 134 | 134 | 134 | 134 | | | | |
| Substance-Related Problems (T2) | r | 0.098 | 0.059 | 0.016 | .804** | .587** | 1 | | | |
| | p-value | 0.286 | 0.522 | 0.859 | <0.001 | <0.001 | | | | |
| | N | 120 | 120 | 120 | 120 | 120 | 120 | | | |
| Degree of Use (T2) | r | 0.040 | 0.075 | -0.027 | .631** | .838** | .650** | 1 | | |
| | p-value | 0.662 | 0.413 | 0.766 | <0.001 | <0.001 | <0.001 | | | |
| | N | 120 | 120 | 120 | 120 | 120 | 120 | 120 | | |
| TIV | r | .666** | .787** | .787** | 0.062 | 0.055 | 0.159 | 0.129 | 1 | |
| | p-value | <0.001 | <0.001 | <0.001 | 0.475 | 0.530 | 0.083 | 0.161 | | |
| | N | 134 | 134 | 134 | 134 | 134 | 120 | 120 | 134 | |
| age | r | -.204* | -0.097 | -0.145 | -0.116 | -.104 | -.187* | -0.100 | 0.130 | 1 |
| | p-value | 0.018 | 0.262 | 0.094 | 0.182 | 0.230 | 0.041 | 0.279 | 0.135 | |
| | N | 134 | 134 | 134 | 134 | 134 | 120 | 120 | 134 | 134 |

## References


[1] Hildebrandt MK, Dieterich R, Endrass T. Disentangling substance use and related problems: urgency predicts substance-related problems beyond the degree of use. BMC Psychiatry 2021;21:1–10. https://doi.org/10.1186/S12888-021-03240-Z/TABLES/3.

[2] Moffat AC, Osselton MD, Widdop B. Clarke's Analysis of Drugs and Poisons in Pharmaceticals. London Pharmaceutical 2011:1–2736.

[3] Beesdo-Baum K, Zaudig M, Wittchen H-U. SCID-5-CV Strukturiertes Klinisches Interview für DSM-5® - Störungen - Klinische Version. Deutsche Bearbeitung des Structured Clinical Interview for DSM-5® - Clinician Version von Michael B. First, Janet B.W. Williams, Rhonda S. Karg, Robert L. Spitzer 2019.

[4] Hildebrandt MK, Schwarz K, Dieterich R, Endrass T. Dissociating the Link of Neural Correlates of Inhibition to the Degree of Substance Use and Substance-Related Problems: A Preregistered, Multimodal, Combined Cross-sectional and Longitudinal Study. Biol Psychiatry 2023;94:898–905. https://doi.org/10.1016/J.BIOPSYCH.2023.06.017.

[5] Gaser C, Dahnke R, Thompson PM, Kurth F, Luders E, Initiative ADN. CAT – A Computational Anatomy Toolbox for the Analysis of Structural MRI Data. BioRxiv 2023:2022.06.11.495736. https://doi.org/10.1101/2022.06.11.495736.

[6] Rajapakse JC, Giedd JN, Rapoport JL. Statistical approach to segmentation of single-channel cerebral mr images. IEEE Trans Med Imaging 1997;16:176–86. https://doi.org/10.1109/42.563663.

[7] Tohka J, Zijdenbos A, Evans A. Fast and robust parameter estimation for statistical partial volume models in brain MRI. Neuroimage 2004;23:84–97. https://doi.org/10.1016/J.NEUROIMAGE.2004.05.007.

[8] Ashburner J. A fast diffeomorphic image registration algorithm. Neuroimage 2007;38:95–113. https://doi.org/10.1016/J.NEUROIMAGE.2007.07.007.

[9] Malone IB, Leung KK, Clegg S, Barnes J, Whitwell JL, Ashburner J, et al. Accurate automatic estimation of total intracranial volume: A nuisance variable with less nuisance. Neuroimage 2015;104:366. https://doi.org/10.1016/J.NEUROIMAGE.2014.09.034.

[10] Zhang M, Gao X, Yang Z, Wen M, Huang H, Zheng R, et al. Shared gray matter alterations in subtypes of addiction: a voxel-wise meta-analysis. Psychopharmacology (Berl) 2021;238:2365–79. https://doi.org/10.1007/S00213-021-05920-W.

[11] Alemán-Gómez Y. M-GL, V-HernandezP. IBASPM: Toolbox for automatic parcellation of brain structures, Florence, Italy: Presented at the 12th Annual Meeting of the Organization for Human Brain Mapping; 2006.

[12] Hall MG, Alhassoon OM, Stern MJ, Wollman SC, Kimmel CL, Perez-Figueroa A, et al. Gray matter abnormalities in cocaine versus methamphetamine-dependent patients: a neuroimaging meta-analysis. Am J Drug Alcohol Abuse 2015;41:290–9. https://doi.org/10.3109/00952990.2015.1044607.

[13] Xiao PR, Dai ZY, Zhong JG, Zhu YL, Shi HC, Pan PL. Regional gray matter deficits in alcohol dependence: A meta-analysis of voxel-based morphometry studies. Drug Alcohol Depend 2015;153:22–8. https://doi.org/10.1016/J.DRUGALCDEP.2015.05.030.

[14] Klaming R, Harlé KM, Infante MA, Bomyea J, Kim C, Spadoni AD. Shared gray matter reductions across alcohol use disorder and posttraumatic stress disorder in the anterior cingulate cortex: A dual meta-analysis. Neurobiol Stress 2018;10. https://doi.org/10.1016/J.YNSTR.2018.09.009.

[15] Yan H, Xiao S, Fu S, Gong J, Qi Z, Chen G, et al. Functional and structural brain abnormalities in substance use disorder: A multimodal meta-analysis of neuroimaging studies. Acta Psychiatr Scand 2023;147:345–59. https://doi.org/10.1111/ACPS.13539.

[16] Pando-Naude V, Toxto S, Fernandez-Lozano S, Parsons CE, Alcauter S, Garza-Villarreal EA. Gray and white matter morphology in substance use disorders: a neuroimaging systematic review and meta-analysis. Translational Psychiatry 2021 11:1 2021;11:1–18. https://doi.org/10.1038/s41398-020-01128-2.

[17] Hill-Bowen LD, Riedel MC, Salo T, Flannery JS, Poudel R, Laird AR, et al. Convergent gray matter alterations across drugs of abuse and network-level implications: A meta-analysis of structural MRI studies. Drug Alcohol Depend 2022;240:109625. https://doi.org/10.1016/J.DRUGALCDEP.2022.109625.

[18] Ersche KD, Williams GB, Robbins TW, Bullmore ET. Meta-analysis of structural brain abnormalities associated with stimulant drug dependence and neuroimaging of addiction vulnerability and resilience. Curr Opin Neurobiol 2013;23:615–24. https://doi.org/10.1016/J.CONB.2013.02.017.

[19] Rolls ET, Huang CC, Lin CP, Feng J, Joliot M. Automated anatomical labelling atlas 3. Neuroimage 2020;206:116189. https://doi.org/10.1016/J.NEUROIMAGE.2019.116189.

[20] Eickhoff SB, Stephan KE, Mohlberg H, Grefkes C, Fink GR, Amunts K, et al. A new SPM toolbox for combining probabilistic cytoarchitectonic maps and functional imaging data. Neuroimage 2005;25:1325–35. https://doi.org/10.1016/J.NEUROIMAGE.2004.12.034.
